# *Momordica charantia* (bitter melon) efficacy and safety on glucose metabolism in Korean prediabetes participants: a 12-week, randomized clinical study

**DOI:** 10.1007/s10068-022-01214-9

**Published:** 2022-12-14

**Authors:** Bukyung Kim, Hak Sung Lee, Hye-Jin Kim, Hyolynn Lee, In-young Lee, Soyoung Ock, Sukyoung Kwon, Sang-Soo Kang, Youngsik Choi

**Affiliations:** 1 Department of Internal Medicine, Kosin University School of Medicine, Busan, Korea; 2 Natural Product Research Team 2, Skin & Natural Products Lab., Kolmar BNH Co., Ltd., Seoul, Korea; 3 Department of Internal Medicine, Gyeongsang National University Hospital, Gyeongsang National University School of Medicine, Jinju, Korea; 4 Department of Anatomy and Neurobiology, Institute of Health Science, Gyeongsang National University School of Medicine, Jinju, Korea; 5 Department of Internal Medicine, Kosin University College of Medicine, 262 Gamcheonro, Seogu, Busan, 602-702 Korea

**Keywords:** Bitter melon, *Momordica charantia*, Glucose, Prediabetes

## Abstract

This study was performed to investigate the effects of bitter melon extract (BME) on glucose metabolism, insulin resistance, and various metabolic parameters of participants with prediabetes. A 12-week randomized placebo-controlled clinical study was conducted with prediabetic patients. A total of 76 participants were randomly assigned to initiate the study. In the final analysis, 33 and 32 subjects were included in the BME and placebo groups, respectively. Results showed that 75 g oral glucose tolerance test (OGTT) blood glucose level decreased in BME group after 12 weeks. The glucose level after 30 min of glucose ingestion decreased significantly. The glucagon level in the BME group after 12 weeks significantly decreased 120 min after 75 g OGTT. These results suggested that bitter melon exhibits glucose-lowering effects through suppression of glucagon levels in people with prediabetes.

## Introduction

Bitter melon (*Momordica charantia* L.) is mainly found in subtropical regions, such as China, India, Thailand, East Africa, and Latin America. Balsam pear, carilla, cerasee, cundeamor, goo-fah, and karela are its alternate names. It is called bitter gourd or bitter melon because of its bitter taste (Tahira and Hussain, 2014). Developing countries such as Brazil, China, Colombia, Cuba, Ghana, and India has used it traditionally as a treatment for diabetes. It is also applied to treat local wounds (Raman and Lau, 1996). It has antibacterial, antiviral, anticancer, and anti-inflammatory effects. It has been also used for gastric ulcer diseases (Grover et al., 2002; Rathi et al., 2002). Many patients with diabetes use bitter melon as alternative medicine in Eastern culture. Many patients in Korea believe that bitter melon has glucose-lowering effects.

Bitter melon seeds, leaves, berries, and fruit peels (Singh et al., 2011) has about 228 components. It is rich in vitamins A, B1, B2, B9, C, and E and minerals such as calcium, potassium, zinc, magnesium, phosphorus, and iron. It has abundant bioactive substances, including anthraquinones, essential oil, saponin, triterpenes, alkaloids, and momordicine (Joseph and Jini, 2013; Kenny et al., 2013; Liu et al., 2009). Blood glucose regulation is linked to the following substances: charantin (a mixture of steroidal saponins), polypeptide-p, vicine, and momordin analogs (e.g., momordinol, momordicilin, momorcharin, and momordicin). Polypeptide-p is an insulin-like protein also called p- insulin or v-insulin (Oliveira et al., 2018). Their fasting blood glucose levels decrease (Habicht et al., 2014; Krawinkel and Keding, 2006) when purified polypeptide-p extracted from bitter melon is injected subcutaneously in patients with type 1 and type 2 diabetes. Glucose clearance increases when terpene-based substances in bitter melon, momodicoside S and momodicoside T, are parenterally administered to healthy mice. Another substance in bitter melon, trehal, suppresses *α*-glucosidase activities by 40% (Khatib et al., 2017).

Body weight, body fat mass, and plasma lipid levels (cholesterol, insulin, glucagon, and C-peptide) decreased when administrating bitter melon extract (BME) orally in an animal model with high-fat diet (HFD)-induced diabetes (Yoon et al., 2017); SIRT1, AMPK, and PPARα factors related to glucose consumption increased, whereas factors associated with fat accumulation such as SREBP1c decreased significantly. It was shown that glucagon secretion significantly decreased when oral BME administration on HFD/streptozotocin (STZ)-induced diabetes in mice (Kim et al., 2020). Their blood glucose levels were suppressed 15 min after 75 g OGTT in the BME administration group.

Therefore, we hypothesized that BME can improve in humans with impaired glucose metabolism at prediabetes. This study was conducted to investigate blood glucose-lowering effects and mechanisms of bitter melon, and to observe the safety of BME in Korean participants with prediabetes.

## Materials and methods

### Study design

This study was designed as a 12-week, randomized clinical trial. All participants were randomly assigned a sequence, and double-blinded to the participant and investigator until the study was completed.

Participants who had prediabetes (100–125 mg/dL fasting blood glucose or 5.6–6.4% in HbA1C) and were aged 18–80 years were enrolled. Informed consent from all participants were obtained at Kosin University Hospital (Busan, Korea). This study was approved by the Kosin University College of Medicine Institutional Review Board (IRB No. 1040549-201603-BM-004).

**Fig. 1:**
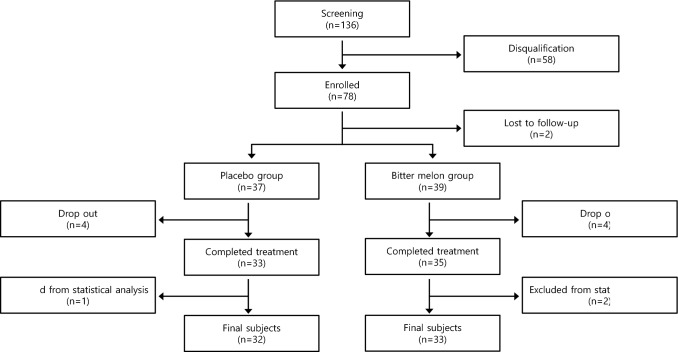
Research progression flowchart

The exclusion criteria were as follows: (1) patients suffering from other serious diseases (cancer, cirrhosis, chronic hepatitis, or kidney disease), exhibiting hepatic GOT/GPT 80 IU/L and serum creatinine higher than 2.0 mg/dL, or having dialysis, (2) patients with cancer, (3) patients with type 1 diabetes and patients using steroids within 6 months or drugs that may affect blood glucose levels, (4) patients participating in other clinical trials, (5) patients experiencing a serious adverse reaction or requesting test discontinuation due to an adverse reaction, and (6) patients showing less than 80% compliance with the test product. The progress of the study is shown in a flowchart (Fig. 1).

### Test supplement

Kolmar BNH (Sejong, Korea) prepared BME supplements, a product named “SugarKatcher S52.” The dried unripe bitter gourd was extracted with 70% ethanol at 70 °C, the conditions at which BME showed the highest antioxidant power as well as α-glucosidase and pancreatic lipase inhibition activities; subsequently, it was spray-dried for powder preparation. At this condition, the concentrations of total polyphenol and total flavonoid were the highest, as represented by gallic acid (7.66 mg/g) and tannin acid (7.77 mg/g; Moon et al., 2015); another index component—γ-aminobutyric acid (GABA)—1.08 mg/g (Lee et al., 2018). Cellulose, maltodextrin, hydroxypropyl methylcellulose, and a pigment matching the BME dose were the main contents of the placebo tablets. All participants were instructed to consume two tablets three times daily (2.4 g/day). The BME and placebo capsules were packaged in an indistinguishable manner and marked with numbers assigned to the participant.

### Measurement of blood glucose parameters

The effectiveness of the BME supplement was evaluated by measuring the following parameters: fasting glucose, HbA1C, insulin, glucagon, C-peptide, lipid profiles; total cholesterol (TC), high-density lipoprotein cholesterol (HDL-C), low-density lipoprotein cholesterol (LDL-C), and triglyceride (TG) after 12 weeks of administration. OGTT was performed after 8 h of fasting. Oral ingestion of 75 g of glucose, fasting glucose, insulin, c-peptide, and glucagon were measured at baseline, 30 min, 60 min, and 120 min, respectively.

The hexokinase method was used to analyze blood glucose. The AD2400 (Siemens, Germany) was used to measure TC, HDL-C, LDL-C, and TG, whereas ADVIA Centaur XPT (Siemens, Germany) was used to determine insulin and C-peptide.

Insulin resistance was represented by homeostatic model assessment for insulin resistance (HOMA-IR), and insulin secretion was represented by HOMA-β (Wallace et al., 2004) and insulinogenic index (Singh and Saxena, 2010). Insulin sensitivity also calculated using the Matsuda index (Matsuda and DeFronzo, 1999). These were calculated from the OGTT values.

### Statistical analysis

SPSS ver. 19 (IL, USA) was used to analyze data. BME and placebo groups were comparatively analyzed via Student’s *t*-test. A chi-square test compared the safety events between the two groups. We used the paired t-test when comparing the values before and after administration in each group. Data were expressed as mean standard deviation, and data with p < 0.05 were considered statistically significant.

## Results and discussion

### Participants

A total of 58 participants were disqualified out of the 136 that were screened, and a total of 78 were enrolled in this study. About two participants were lost to follow-up after being enrolled; the 76 remaining participants were randomly assigned to the BME group (n = 39) and the placebo group (n = 37). Among them, four participants dropped out of the BME group, and four participants withdrawal of the placebo group. In the BME group, two of the four who dropped out could not be contacted without any special reason; one was hospitalized but did not disclose the reason for the hospitalization, whereas the other one was discontinued given abandonment of participation. In the placebo group, three out of four who dropped out could not be contacted without any specific reason, and one withdrawal due to abandonment of participation. Only one from the placebo group and two from the BME group were excluded from the analysis due to lack of compliance at the end of the 12-week study. Finally, 32 placebo and 33 BME participants were analyzed, respectively.

There was no significant difference at the baseline characteristics, and randomization can be confirmed well (Table 1).

**Table 1:** Baseline clinical characteristics of all participants

	Placebo (*n* = 32)	Bitter melon (*n* = 33)	*p* value
Sex (M:F)	16:16	10:23	< 0.001
Age	53.6 ± 7.6	56.7 ± 11.3	0.290
Height (cm)	164.0 ± 7.6	161.9 ± 7.9	0.306
Weight (kg)	65.1 ± 11.4	63.2 ± 9.1	0.401
Abdominal circumference (cm)	85.6 ± 8.1	85.6 ± 8.3	0.807
Systolic BP (mmHg)	127.3 ± 10.8	126.3 ± 11.9	0.676
Diastolic BP (mmHg)	71.7 ± 8.9	73.1 ± 8.6	0.957
Fasting glucose (mg/dL)	107.1 ± 19.6	101.1 ± 12.3	0.768
HbA1c (%)	5.9 ± 0.4	5.9 ± 0.3	0.720
GOT (IU/L)	25.5 ± 6.9	29.0 ± 11.5	0.138
GTP (IU/L)	23.9 ± 11.7	24.4 ± 12.5	0.848
ALP (IU/L)	64.0 ± 32.1	64.0 ± 19.7	0.996
rGTP (IU/L)	40.1 ± 77.4	27.5 ± 23.3	0.351
Total cholesterol (mg/dL)	185.8 ± 31.3	195.1 ± 36.1	0.267
HDL-C (mg/dL)	52.5 ± 14.4	58.4 ± 16.2	0.463
LDL-C (mg/dL)	111.6 ± 26.1	117.0 ± 32.5	0.124
Tg (mg/dL)	158.9 ± 201.6	130.6 ± 78.0	0.456

### Determination of blood glucose levels

**Fig. 2:**
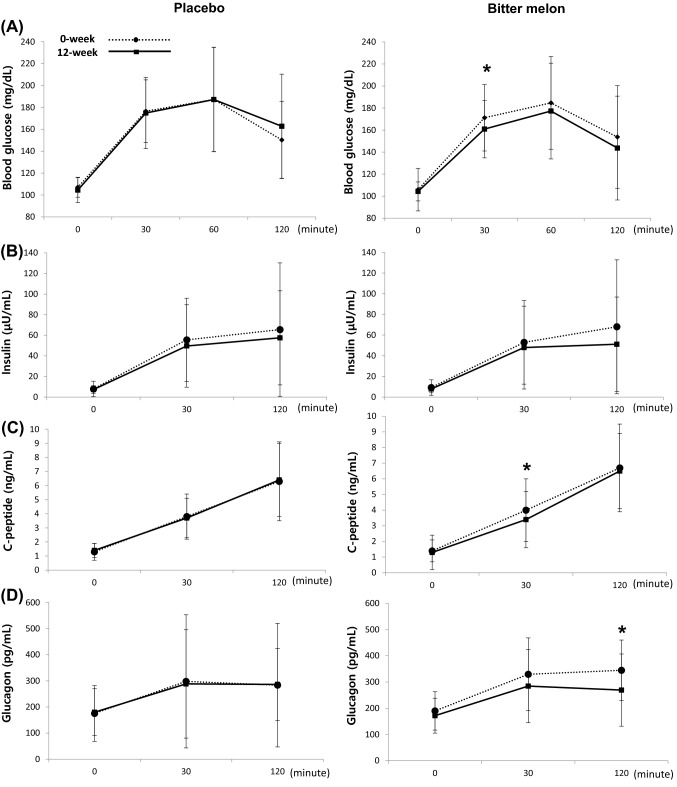
Comparison of the results in 75 g OGTT (Oral Glucose Tolerance Test) between 0 week and after 12 weeks **A** Blood glucose, **B** Insulin, **C** C-peptide, **D** Glucagon levels supplementation with placebo and Bitter Melon Extract (BME) group. It was indicated comparison before and after the test within the same group, *p < 0.05

Currently used as a diagnostic criterion for diabetes, fasting blood glucose was created by the American Diabetes Association in 1997 to predict the occurrence of retinopathy, a microvascular complication of diabetes. A 2-h glucose level of 75 g OGTT is one of the diagnostic criteria for diabetes when 200 mg/dL or higher (James et al., 1997). OGTT reveals how insulin regulates blood glucose in the glucose metabolic process obtained from external sources, such as a meal (Nathan et al., 2007). Blood glucose levels were measured via a 75 g OGTT in this study. The blood glucose levels after 12 weeks in the BME group were lower than before treatment, and the 30 min time point was significantly lower than baseline (p < 0.05). The blood glucose levels did not change from the baseline and 12 weeks after OGTT in the placebo group (Fig. 2A). The differences between baseline and 12-week glucose levels were significantly different at the 120 min time point (Table 2).

**Table 2:** Measurement differences in biomarker values before and after treatment

		Placebo (*n* = 32)	Bitter melon (*n* = 33)	*p* value
Blood glucose (mg/dL)	0 min	− 2.53 ± 3.47	− 1.03 ± 1.32	0.684
	30 min	− 1.59 ± 4.17	− 10.30 ± 5.14	0.194
	60 min	− 0.1 ± 40.3	− 7.4 ± 27.6	0.396
	120 min	12.50 ± 8.27	− 10.03 ± 6.18	0.032*
	C_max_	4.63 ± 5.64	− 8.91 ± 4.55	0.070
	AUC	286 ± 3229	− 956 ± 2210	0.076
Insulin (μU/mL)	0 min	− 0.61 ± 1.17	− 1.39 ± 1.31	0.659
	30 min	− 5.97 ± 9.26	− 4.98 ± 5.10	0.925
	120 min	− 7.87 ± 8.86	− 23.95 ± 11.32	0.269
	C_max_	− 8.41 ± 10.55	− 19.05 ± 10.14	0.470
C-peptide (ng/mL)	0 min	0.149 ± 0.201	− 0.021 ± 0.086	0.435
	30 min	− 0.092 ± 0.289	− 0.519 ± 0.207	0.231
	120 min	0.130 ± 0.457	− 0.205 ± 0.378	0.573
	C_max_	0.13 ± 0.40	− 0.29 ± 0.36	0.443
Glucagon (pg/mL)	0 min	4.57 ± 11.45	− 18.17 ± 11.67	0.170
	30 min	− 9.49 ± 20.28	− 92.17 ± 44.70	0.101
	120 min	2.59 ± 16.56	− 75.48 ± 32.75	0.039*
	C_max_	− 8.3 ± 18.6	− 112.1 ± 45.0	0.039*
HOMA-IR		− 0.292 ± 1.490	− 0.153 ± 1.200	0.865
HOMA-β		− 0.393 ± 1.949	− 0.310 ± 2.023	0.674
Matsuda index		0.33 ± 4.36	− 0.03 ± 5.82	0.77
Insulinogenic index		− 0.106 ± 1.122	− 0.281 ± 1.378	0.571

The differences were calculated based on after clinical trial (12 weeks) values minus baseline (0 weeks) values

*C*_*max*_ the maximum concentration, *AUC* area under curve

*p < 0.05

Unfortunately, we could not observe the fasting blood glucose change after 12 weeks, because the participants were not diabetic. Therefore, their baseline fasting blood glucose levels were not high enough to show the fasting glucose-lowering effect of the BME supplement for 12 weeks.

### Insulin resistance and secretion determination

There was no significant difference in insulin resistance with HOMA-IR and insulin secretion using HOMA-β, and insulinogenic index at baseline and 12 weeks. Differences between baseline and 12 weeks were not different in both groups (Table 2).

### Determination of insulin levels

Insulin regulates the overall metabolism of the body, and participates in carbohydrate, fat, and protein metabolism. Its most important function is reducing glucose levels in the blood in glucose metabolism. Insulin promotes and inhibits glycogen synthesis in the liver, promotes glycogen synthesis, increases triglyceride synthesis, and inhibits gluconeogenesis. It also accelerates protein synthesis and glycogen synthesis in muscles. It enhances triglycerides storage, and reduces blood glucose concentrations in adipose tissues (Withers and White, 2000).

Fasting insulin and insulin 30 min and 120 min after 75-g OGTT decreased after 3 months of bitter melon administration in the BME group, but the baseline changes were not statistically significant. Insulin also decreased for fasting in the placebo group, 30 min, and 120 min from baseline after 3 months of placebo. These differences were also not statistically significant (Fig. 2B).

### Determination of C-peptide levels

Insulin is synthesized in the form of proinsulin in the beta cells of the pancreas, and C-peptide is a connecting peptide that exists between the insulin *α*- and *β*-chains. The C-peptide portion is hydrolyzed and released after proinsulin is transported into secretory vesicles. Insulin is formed when the separated *α*- and *β*-chains are combined; when insulin is secreted, C-peptide is also secreted. Therefore, endogenous insulin and C-peptide exist in blood at the same ratio (Kuzuya et al., 1976; Polonsky, 1995).

The C-peptide levels during fasting at 30 min and 120 min after 75 g OGTT decreased after 12 weeks of BME supplementation. The C-peptide level 30 min after 75 g OGTT was significantly reduced (p < 0.01). This was not attributable to decreased beta cell function, but to a lowered insulin requirement after 12 weeks following BME administration, due to a significant decrease in blood glucose at the 30 minute interval. The fasting C-peptide levels increased after the participants received the placebo in the placebo group, but this increase was not statistically significant (Fig. 2C).

### Determination of glucagon levels

Glucagon and insulin play an important role in maintaining glucose homeostasis and pre-meal glucose concentrations in the normal range by producing glucose in the fasting state. Glucagon secretion is stimulated when blood glucose decreases, whereas it is inhibited by hyperglycemia and insulin secretion (Gosmanov et al., 2005).

The glucagon level in the BME group decreased after 12 weeks after 75 g OGTT in this study. The glucagon level 120 min after OGTT was significantly decreased after 12 weeks compared with baseline (p < 0.05). However, there were no differences in glucagon levels after 12 weeks compared with the baseline in the placebo group (Fig. 2D). The maximum concentration of a serum biomarker is referred to as C_max_. The maximal concentration of glucagon level decreased significantly (Table 2).

We could observe significant glucose-lowering effects of BME through the OGTT in this study. However, no significant differences in insulin secretion and resistance were determined. An interesting and unique result revealed in this study was the change in glucagon levels. The glucagon level significantly reduced after an oral glucose load was administered in the BME group.

An altered insulin-to-glucagon ratio has been shown in previous studies in patients with diabetes (Unger et al., 1972). Glucose regulation is impaired by an increase in fasting glucagon and suppression of the required glucagon prolongation at the prediabetes stage before the onset of diabetes (Kim et al., 2020). Glucagon is stimulated by low glucose levels, therefore postprandial glucagon is suppressed. This study showed that postprandial glucagon was not sufficiently suppressed in prediabetes and BME can lower it.

### Determination of safety of BME

Fatigue, dizziness, and pruritus were the most commonly reported side effects. Fatigue and pruritus were more common in the test group. Dizziness was reported only in the test group. Also, three people in each group experienced cough, low blood pressure, weight gain, and dyspepsia. There was one acute hepatitis case in the test group. The disease was confirmed to have occurred after taking herbal medicine 2 weeks before the last blood sampling during the test period. Hence, the person was excluded from the analyses. There were no critical side effects in either group (Table 3).

**Table 3:** Safety events from all participants in this study

	Placebo (n = 37)	Bitter Melon (n = 39)	p value
Fatigue	4	11	0.084
Dizziness	0	8	0.005
Reduced pain	1	0	0.487
Vaginitis	0	1	0.194
Periodontitis	0	1	1.000
Colon polyp	1	0	1.000
Weight gain	6	3	0.610
Bronchitis	1	1	0.487
Itching sense	1	5	0.303
Urinary frequency	0	2	1.000
Dyspepsia	4	1	0.201
Dehydration	0	1	0.494
Gastritis	0	1	0.194
Uterine cyst	1	0	1.000
Weight loss	0	2	1.000
Acute hepatitis	0	1	0.487
Anemia	1	0	0.494

Although comas and convulsions in children due to hypoglycemia and paroxysmal atrial fibrillation have been reported, no toxic side effects have been reported previously including death. Bitter melon is also contraindicated during pregnancy because of the high risk of miscarriage (Basch et al., 2003). Therefore, children and pregnant women should not take BME.

There are some limitations in this study. First, given that the participants were not diabetic, their basal blood glucose was not high enough to observe the effect of 12 weeks of BME administration. They were healthy, and it was difficult to identify any difference even after 12 weeks, because the baseline liver function test levels and lipid profile levels were all within the normal range. Second, due to the high number of unexpected dropouts, the number included in the final analysis was small. Nevertheless, the BME group showed a significant decrease in blood glucose and significant suppression of glucagon following the OGTT.

In conclusion, Korean participants with prediabetes who were given BME are expected to improve their postprandial glucose levels possibly because of glucagon suppression. In the future, a long-term cohort study should be performed to confirm the prophylactic effects of bitter melon in diabetes, especially prediabetes.
